# Image-guided percutaneous revascularization of the coronary arteries

**DOI:** 10.1093/ehjimp/qyae122

**Published:** 2024-11-23

**Authors:** Mirvat Alasnag, Fawaz Bardooli, Tom Johnson, Alexander G Truesdell

**Affiliations:** Cardiac Center, King Fahd Armed Forces Hospital, PO Box 126418, Jeddah 21372, Saudi Arabia; Department of Cardiovascular, Mohammed Bin Khalifa Cardiac Centre, Riffa, Bahrain; Department of Cardiology, Bristol Royal Infirmary, Bristol, UK; Heart and Vascular Center, Virginia Heart/Inova Schar Heart and Vascular, Falls Church, VA, USA

**Keywords:** intravascular ultrasound, optical coherence tomography, cardiovascular outcomes

## Abstract

The European Society of Cardiology recently updated guidelines on the management of chronic coronary syndromes upgrading the use of intracoronary imaging for complex percutaneous coronary interventions (PCI) to a class 1A recommendation. It is essential that the interventional community appreciate the additive value of intracoronary imaging over angiography alone—not only to obtain optimal acute PCI results but also to improve longer-term cardiovascular outcomes. The purpose of this manuscript is to review the latest evidence that informed the recent guideline recommendations and expand on the specific role of the different imaging modalities before, during, and after PCI.

## Graphical Abstract

**Graphical Abstract:**
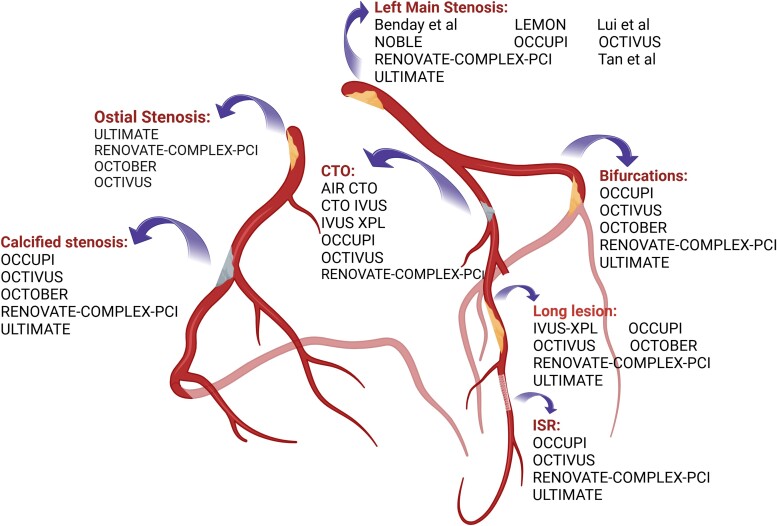
Central figure summarizing the current body of evidence.

## Introduction

With modern drug-eluting stent (DES) technologies, the rates of target-vessel revascularization (TVR), TLR (target lesion revascularization), TLF (target lesion failure), and TVF (target vessel failure) have improved considerably.^1^ Recently published data on image-guided PCI with intravascular ultrasound (IVUS) and optical coherence tomography (OCT) consistently demonstrated significantly reduced major adverse cardiovascular event (MACE) rates. As such, the European Society of Cardiology (ESC) guidelines appropriately newly assigned a class 1A recommendation to IVUS and OCT for complex lesion subsets.^2^ Although there is no standardized definition for ‘complex’ lesions, contemporary clinical trials typically include lesions such as left main coronary artery, bifurcations, long lesions, calcified lesions, in-stent restenosis, and chronic total occlusion (CTO).^3^ The ESC guidelines have restricted it to left main (LM) disease, bifurcations, and long lesions. Intravascular imaging (IVI) permits accurate identification of plaque morphology (to include identification and characterization of calcium), landing zones, and stent sizing before embarking upon PCI. It also allows operators to determine whether the plaque modification is adequate prior to definitive stenting. Finally, acceptable stent apposition and expansion (and achievement of appropriate endpoints and exclusion of complications) can be assessed through imaging. This review will summarize the role of imaging in the different stages of PCI as well as the contemporary, large, body of evidence that drove the recent class 1A guideline upgrade (*Graphical Abstract*).^4–7^

## Contemporary evidence

### ILUMIEN IV

ILUMIEN IV (Optical Coherence Tomography-Guided versus Angiography-Guided PCI) was a prospective trial where patients with medication-treated diabetes or complex coronary artery lesions were randomly assigned to undergo OCT-guided PCI or angiography-guided PCI. A final blinded OCT procedure was performed in patients in the angiography group. The two primary efficacy endpoints were the minimum stent area after PCI as assessed with OCT and TVF at 2 years, defined as a composite of death from cardiac causes, target vessel-related myocardial infarction (TV-MI), or ischaemia-driven TVR. The trial was conducted in 18 countries. A total of 2487 patients were included, with 1233 patients in the OCT-guided PCI arm and 1254 angiography-guided PCI arm. The minimum stent area after PCI was 5.72 ± 2.04 mm^2^ in the OCT group and 5.36 ± 1.87 mm^2^ in the angiography group (mean difference, 0.36 mm^2^; *P* < 0.001). TVF occurred in 88 patients in the OCT group and in 99 patients in the angiography group (*P* = 0.45). Adverse events occurred in one patient in the OCT group and in two patients in the angiography group. Stent thrombosis (ST) by 2 years occurred in 0.5% in the OCT group and in 1.4% in the angiography group. Although there was no difference in TVF at 2 years, OCT guidance resulted in a larger minimum stent area and a significant reduction in ST.^8^ It should be noted that the treatment assignment was not concealed from the participating operators and the impact of the individual interventional cardiologists’ experience and skills was not accounted for. Furthermore, longer follow-up may be necessary to determine the incidence of important clinical endpoints. As such, despite a slightly larger minimum stent area with OCT guidance, TVF was not significantly different.

### RENOVATE-COMPLEX-PCI Trial

The purpose of the RENOVATE-COMPLEX-PCI Study (Randomized Controlled Trial of IVI Guidance versus Angiography-Guidance on Clinical Outcomes after Complex Percutaneous Coronary Intervention) was to investigate whether IVI-guided PCI with the use of IVUS or OCT would improve clinical outcomes compared with angiography-guided PCI in complex coronary artery lesions.^9^ This was a prospective, multicentre, open-label trial conducted in South Korea. A total of 1639 patients were randomized, where 1092 were assigned to imaging-guided PCI and 547 to angiography-guided PCI. The choice between intravascular ultrasonography and OCT was at the operators’ discretion. The primary endpoint was a composite of death from cardiac causes, TV-MI, or clinically driven TVR. The median follow-up was 2.1 years, at which time the primary endpoint occurred in 7.7% of the imaging group and 12.3% of the angiography group (*P* = 0.008). Death from cardiac causes occurred in 1.7% of the imaging group and 3.8% of the angiography group; TV-MI in 3.7% and 5.6%, respectively; and clinically driven TVR in 3.4% and 5.5%, respectively. RENOVATE-COMPLEX-PCI conclusively demonstrated that IVI-guided PCI led to a lower risk of a composite of death from cardiac causes, TV-MI, or clinically driven TVR in complex coronary artery lesions.^9^ It is important to recognize that a considerable proportion of the cohort were recruited in a single centre with highly experienced operators that may have partially driven the better outcomes. Finally, we should acknowledge nuanced differences in complex procedures, whereby, for example, CTO and ostial diseases are better suited for IVUS vs. OCT. Interestingly, there was a positive impact on cost-efficiency; however, this was appreciated only after 6 years. A final important consideration is that RENOVATE-COMPLEX-PCI was primarily a single centre trial where significant operator expertise in intracoronary imaging may render the results less generalizable.

### OCTOBER Trial

The OCTOBER Trial (Optical Coherence Tomography Optimized Bifurcation Event Reduction) was a multicentre, open-label, randomized controlled, superiority trial designed to assess clinical outcomes following complex coronary artery bifurcation lesions with OCT guidance compared with angiography-guided PCI. This was an investigator-initiated trial conducted at 38 heart centres in Europe.^10^ Randomization was 1:1, and the primary endpoint was a composite of major adverse cardiac events (MACE), defined as death from a cardiac cause, target lesion MI, or ischaemia-driven TLR at a median follow-up of 2 years. A total of 1201 patients were enrolled (600 in the OCT-guided arm and 601 in the angiography-only arm). Left main PCI constituted 18.5% of the OCT-guided group and 19.3% of the angiography-guided group. At 2 years, the primary endpoint occurred in 10.1% of the OCT-guided group and 14.1% of the angiography-guided group (*P* = 0.035). Procedure-related complications occurred in 6.8% of the OCT-guided group and 5.7% of the angiography-guided group.^10^

A two-stent strategy was performed in 388 patients of the OCT-guided PCI group and 382 patients of the angiography-guided PCI group. This represents a large proportion of the enrolled population and reflects the complexity of disease. Angiographic success was observed in 95.8% of the OCT-guided group and 95.7% of the angiography-guided PCI group. Interestingly, in the OCT-guided PCI, only 4.7% had stent expansion of at least 90%, verified by OCT, in all treated segments. In a substudy of the OCTOBER Trial, unintended stent deformation (USD) was detected in 9.3% of the OCT-guided PCI arm by the core lab.^11^ Abluminal rewiring was identified as the cause in 44% and guide catheter collision in 40%. The USD was corrected in only 54.5% of the PCIs. USD was reported in 18.5% of left main bifurcations. The 2-year major adverse cardiac event rate for patients with untreated USD was 23.3%. These high rates of abluminal wiring and stent deformation emphasize the need for operators to not only obtain but also to appropriately act upon information derived from IVI to optimize acute PCI results and long-term outcomes.^11^ It is also worth noting that crossover to IVUS use in the angiography arm reached 15% in the overall population and for the left main cases, it reached ∼50% of all the cases in the angiography-guided arm, potentially diluting the impact of the OCT-guided approach. Furthermore, there is potential for bias as group assignment was not blinded and operator experience was not reported. There was a marginal advantage to OCT-guided PCI using cluster endpoints at 2 years. A longer follow-up would add clarity with respect to the clinical endpoints.

### IVUS-XPL Trial

The IVUS-XPL Study (Impact of Intravascular Ultrasound Guidance on the Outcomes of Xience Prime Stents in Long Lesions) randomized 1400 patients with long coronary lesions (implanted stent length ≥ 28 mm) to IVUS-guided (*n* = 700) or angiography-guided (*n* = 700) everolimus-eluting stent implantation. Five-year clinical outcomes of those who completed the original trial were reported in 2020 [1183 patients (85%)].^12,13^ The primary outcome was the composite of MACE, including cardiac death, TL-MI, or ischaemia-driven TLR at 5 years. MACE at 5 years occurred in 5.6% of those undergoing IVUS guidance and in 10.7% of those undergoing angiographic guidance (*P* = 0.001). The difference was driven mainly by a lower risk for TLR (4.8% vs. 8.4%; *P* = 0.007). IVUS-guided PCI had significantly lower rate of MACE rates that were not only sustained through 5 years but in fact increased during the follow-up period. This trial also demonstrated that for patients in whom IVUS stent optimization criteria were not met, there was a significantly higher incidence of the primary endpoint at 5 years—further highlighting the importance of leveraging IVI to ensure optimal PCI endpoints.^13^ The value of intracoronary imaging in acute coronary syndrome (ACS), in particular, has been well demonstrated in this study considering that angiographic definition of the disease is suboptimal. Thus, a PCI strategy with appropriate stent sizing and length through IVI impacted procedural outcomes positively as evidenced by a lower MACE rate within 1 year. However, analysis at 5 years noted a non-significant difference in cardiac death and myocardial infarction. Safety issues around aggressive post-dilatation remain unknown at this time.

### ULTIMATE trial

The Ultimate trial (Intravascular Ultrasound Guided Drug Eluting Stents Implantation in All-Comers Coronary Lesions) defined the stent optimization criteria used in most contemporary trials.^14^ Optimal IVUS-guided PCI was defined if three endpoint criteria were met: (i) minimal stent area (MSA) in stented segment > 5.0 mm^2^ or 90% of the minimum lumen area (MLA) at the distal reference segments; (ii) plaque burden at 5 mm proximal or distal to the stent edge < 50%; and (iii) no edge dissection involving media with extension more than 3 mm in length. Suboptimal IVUS-guided PCI was defined if any of the preceding three criteria was not met. The trial included a total of 1448 all comers undergoing DES implantation who were randomly assigned to IVUS or angiographic guidance. The primary endpoint was the TVF (cardiac death, TV-MI, and clinically driven TVR) at 3 years. Only 53% of those enrolled (578 lesions) met all three optimization criteria. The safety endpoint was definite or probable ST.^15^ At 3 years, TVF occurred in 6.6% in the IVUS-guided group and in 10.7% in the angiography-guided group (*P* = 0.01), driven mainly by the decrease in clinically driven TVR (4.5% vs. 6.9%; *P* = 0.05). The rate of definite or probable ST was 0.1% in the IVUS-guided group and 1.1% in the angiography-guided group (*P* = 0.02). An IVUS-defined optimal procedure result was associated with a significant reduction in 3-year TVF compared with a suboptimal procedure result.^15^

### Kim *et al*. (Long Lesion) Trial

In 2013, Kim *et al*. conducted an intention-to-treat analysis for 543 patients treated with stents ≥ 28 mm in length who were randomly assigned to IVUS guidance (*n* = 269) vs. angiography guidance (*n* = 274). The primary endpoint was a composite of MACE including cardiovascular death, MI, TVR, or ST at 1 year.^16^ Total stent length was 32.4 mm in the IVUS-guided arm vs. 32.3 mm in angiography-guided arm. Post-dilation was more frequent in the IVUS-guided arm (54.6% vs. 44.5%, *P* = 0.03). MACE occurred in 4.5% of the IVUS-guided arm and in 7.3% of the angiography-guided arm (*P* = 0.16). However, cross over was high in both arms, so in the per-protocol analysis according to actual IVUS use, minimum stent diameter was larger (2.58 vs. 2.51 mm, *P* = 0.04), and MACE rates were lower: 4.0% in the IVUS-guided arm vs. 8.1% in the angiography-guided arm (*P* = 0.048).^16^

### OCCUPI trial

OCCUPI (optical coherence tomography-guided versus angiography-guided PCI for patients with complex lesions) was an investigator-initiated, multicentre, randomized, open-label, superiority trial conducted in South Korea.^17^ After diagnostic angiography, clinical and angiographic findings were assessed to identify patients who met the criterion of having one or more complex lesions. Patients were randomly assigned 1:1 to receive PCI with OCT guidance (OCT-guidance group) or angiography guidance without OCT (angiography-guidance group). The primary endpoint was major adverse cardiac events (a composite of cardiac death, myocardial infarction, ST, or ischaemia-driven target-vessel revascularization), 1 year after PCI. The primary analysis was done in the intention-to-treat population. The margin used to establish superiority was 1.0 as a hazard ratio. A total of 1604 patients undergoing PCI with everolimus-eluting stents for complex lesions were randomly assigned to receive either OCT-guided PCI (*n* = 803) or angiography-guided PCI (*n* = 801). The median age was 64 years, and 20% were women. The primary endpoint occurred in 5% of 803 patients in the OCT-guided PCI group and 7% of 801 patients in the angiography-guided PCI group [absolute difference −2.8% (95% CI −5.1 to −0.4); hazard ratio 0.62 (95% CI 0.41–0.93); *P* = 0.023]. There was no difference in stroke, bleeding events, and contrast-induced nephropathy.^17^

### Left main trials

In 2017, Andell *et al*.^18^ reported the results of IVUS guidance for LM PCI in both chronic and ACSs from the SCAAR (Swedish Coronary Angiography and Angioplasty Registry). IVUS guidance was used in only 25.2% of the 2468 patients undergoing LM PCI. The IVUS group was younger with more complex lesions. IVUS was associated with larger stent diameters (median, 4 mm vs. 3.5 mm). IVUS was associated with significantly lower incidence of the primary composite endpoint of all-cause mortality, restenosis, or definite ST, and all-cause mortality alone. Following propensity score-matching, IVUS was also associated with a significantly lower rate of the primary endpoint.^18^

Important landmark trials of LM PCI included the NOBLE, Excel, and Main Compare trials. The NOBLE trial randomized patients with LM disease to treatment by PCI or bypass grafting. Of the 603 patients treated using PCI, 435 (72%) underwent post-PCI IVUS assessment with 224 receiving core laboratory analysis. At five years, the composite of major adverse cardiovascular and cerebrovascular events was 18.9% when IVUS was performed vs. 25.0% when no intracoronary imaging was utilized (*P* = 0.45). Although repeat revascularization was not reduced (10.6% vs. 16.5%, *P* = 0.11), LM TLR was reduced (5.1% vs. 11.6%, *P* = 0.01) with IVUS use. The stent optimization criteria in these trials are not clear, and lesion-level analysis may have permitted a better understanding of benefit.^19^

The SYNTAX II study was a multicentre, all-comers, open-label, single arm study that investigated the impact of PCI strategy on clinical outcomes in patients with multivessel disease (454 patients with 1559 lesions). The relationships between post-procedural MSA and lesion-level outcomes at 2 years were reported by Katagiri *et al*. in 2019. A lesion-oriented composite endpoint (LOCE) was defined as the composite of cardiac death, TV-MI, and ischaemia-driven TLR.^20^ A total of 819 lesions with post-procedural MSA were included in the analysis. LOCE was observed in 5.6%, 5.7%, and 3.0% of each tertile (*P* = 0.266). TLR occurred in 5.6%, 4.5%, and 1.5% of each tertile (*P* = 0.042). A smaller post-procedural MSA and lesion SYNTAX Score were independent predictors of TLR on multivariate analysis. The final LM MSA measured on IVUS showed a strong association with adverse events, with three-year MACE almost 20% in the smallest tertile (<8.7mm^2^).^20^

As early as 2005, Fassa *et al*.^21^ demonstrated that the lower range of normal LM MLA was 7.5 mm^2^. LM revascularization was performed in 85.5% of those with an MLA < 7.5 mm^2^ and deferred in 86.9% whose MLA ≥ 7.5 mm^2^. At 3 years, no significant difference in MACE was detected between patients with an MLA < 7.5 mm^2^ who underwent PCI and those with an MLA ≥ 7.5 mm^2^ who were deferred (*P* = 0.28).^21^ de la Torre and colleagues performed a pooled analysis of 1670 patients undergoing DES implantation using IVUS guidance and reported survival free of cardiac death, myocardial infarction, and TLR at 3 years. The use of IVUS is associated with reduced rates of all-cause death and reduced incidence of ST in LMS PCI.^22^ More recently, the Main COMPARE Trial enrolled 975 patients undergoing LM PCI of whom 77.5% had IVUS guidance. At 10 years, the incidence of death (16.4% vs. 31%; *P* < 0.001) and composite of death, Q-wave MI, or stroke was significantly lower in the IVUS-guided arm than angiography-guided arm. The overall benefit of IVUS was consistent across all subgroups (19.2% vs. 32.9%; *P* < 0.001).^23^

### LEMON study

The LEMON study (optical coherence tomography to guide percutaneous coronary intervention of the left main coronary artery) was a prospective, multicentre trial pilot that evaluated outcomes for OCT-guided PCI for mid/distal left main stenosis. The primary endpoint was procedural success defined as residual angiographic stenosis < 50% + TIMI 3 flow in all branches + adequate OCT stent expansion. A total of 70 patients were included with a median age of 72 years. The primary endpoint was achieved in 86% and adequate stent expansion was observed in 86%, significant edge dissection in 30% and residual significant strut malapposition in 24%. The strategy was modified by OCT guidance in 26% of the patients. One-year survival free from MACE was 98.6%.^24^

## Renovate complex PCI Left Main substudy

In the RENOVATE-COMPLEX-PCI, 192 patients had LM disease and were analysed in a prespecified substudy. The primary endpoint was TVF defined as a composite of cardiac death, TV-MI, or clinically driven TVR. At 2 years, imaging-guided PCI was associated with a lower incidence of the primary endpoint compared with angiography-guided PCI (6.8% vs. 25.1%; *P* = 0.010). This was driven by a lower risk of cardiac death or spontaneous TV-MI (1.6% vs. 12.7%; *P* = 0.028). This recent analysis further confirms the critical role of image guidance to improve clinical outcomes in LM PCI, including cardiac death, TV-MI, or TVR.^25^

## Imaging criteria: pre-intervention

**Figure 1:**
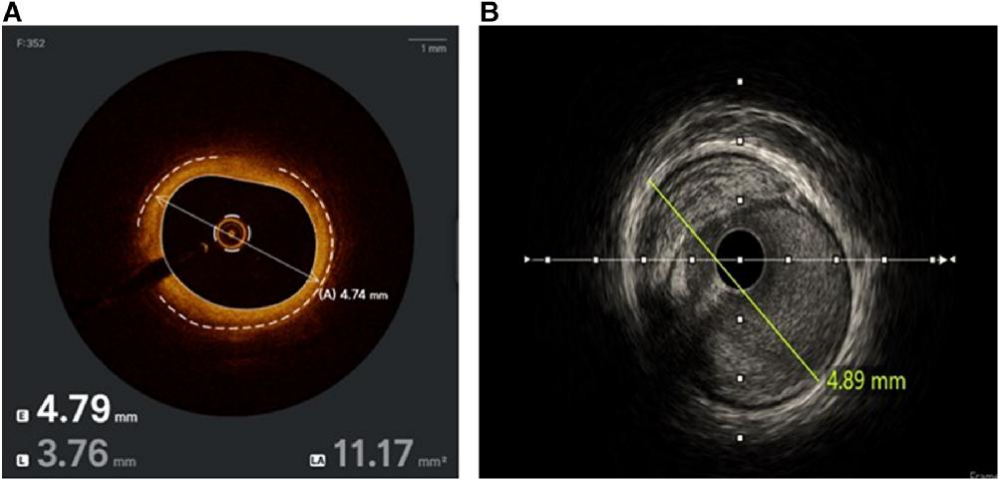
Proper measurement of vessel diameter. (*A*) OCT uses the AI to calculate the EEL as well as well as luminal diameter. (*B*) IVUS image with manual measurement of the vessel diameter.

Both IVUS and OCT can be used before intervention to assess plaque composition or morphology, especially calcified or lipid lesions. This permits the appropriate selection of lesion preparation and plaque modification strategies. In addition, accurate sizing is based on distal vessel reference diameter. Determination of stent length aims for a plaque burden of <50%.^26–30^ Reference diameters for LM arteries generally range between 4.5 ± 0.5 and 3.7 ± 0.4 mm for other non-LM proximal vessel segments.^26^ These may not hold true across ethnic or sex differences. Minimal lumen diameter are generally measured using either the lumen or external elastic lamina (EEL)^27^ (*Figure 1*). Tissue characterization is different for fibrotic, calcific, and thrombotic lesions. More recent iterations of IVUS and OCT software have embedded artificial intelligence-based automated measurements and image interpretation to better guide follow-on lesion preparation and stent strategies.

## Imaging criteria: post-intervention

**Figure 2:**
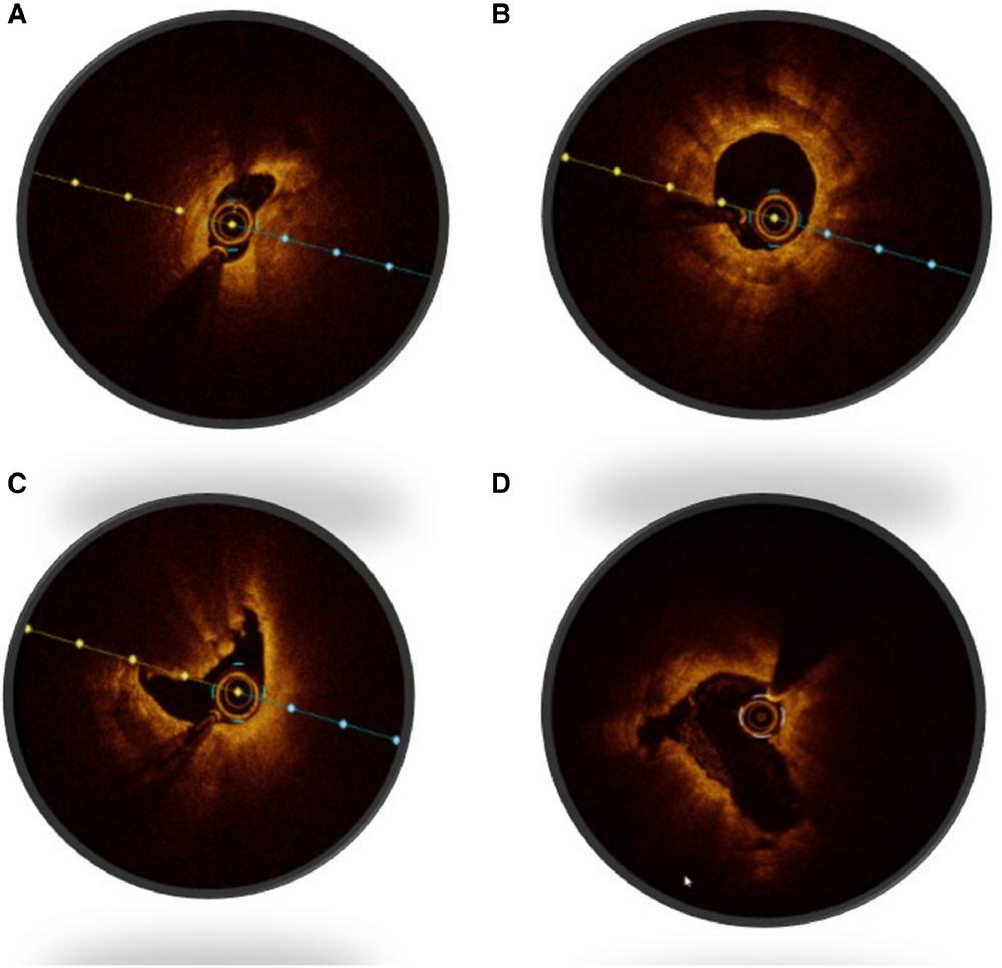
Different patterns of coronary calcification identified by OCT. (*A*) Calcified non-eruptive nodule, (*B*) concentric thick calcium plaque burden, and (*C*) eruptive calcified nodule. (*D*) Fracture of calcified segment after shockwave lithotripsy.

Assessment of post-procedure endpoints (and identification of stent underexpansion, deformation, and malapposition), exclusion of complications (to include edge dissection, haematoma, and tissue protrusion), and confirming the absence of geographic miss and any significant residual inflow or outflow disease by IVI are critical post-intervention steps. MSA and total stent lumen area define acceptable stent expansion. Optimal expansion can be assessed by the use of an absolute value of MSA or percentage stent expansion derived from the MSA and distal or proximal reference areas. The EAPCI consensus statement suggested an absolute OCT MSA > 4.5 mm^2^, and absolute IVUS MSA > 5.5 mm^2^ or relative expansion MSA/average reference lumen > 80%.^27–30^ Although trials such as the CLI-OPCI II registry reported that an absolute MSA of 4.5 mm^2^ can predict device oriented clinical events, absolute values may not account for smaller or larger vessels.^31^ As such, the majority of operators in practice use per cent expansion and apposition especially in non-LM disease. The ADAPT-DES IVUS substudy evaluated criteria set by the ULTIMATE, ILUMIEN III, and IVUS-XPL studies and noted that the ratio of MSA/vessel area at the MSA site was associated with clinical outcomes at 2 years. It is important to acknowledge that this metric can only be generated through IVUS^32^ (*Figure 2*).

**Figure 3:**
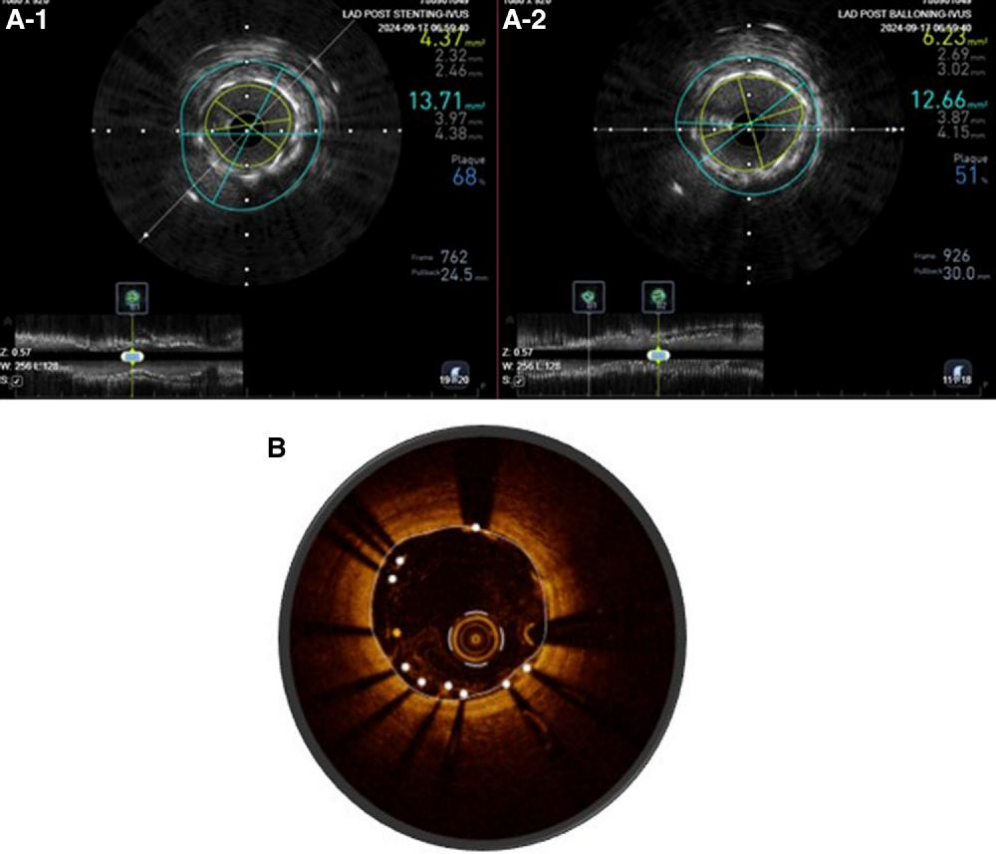
(*A*) Under expanded stent in A-1, and after post-dilatation in A-2. (*B*) IVUS image with malapposed stent.

**Figure 4:**
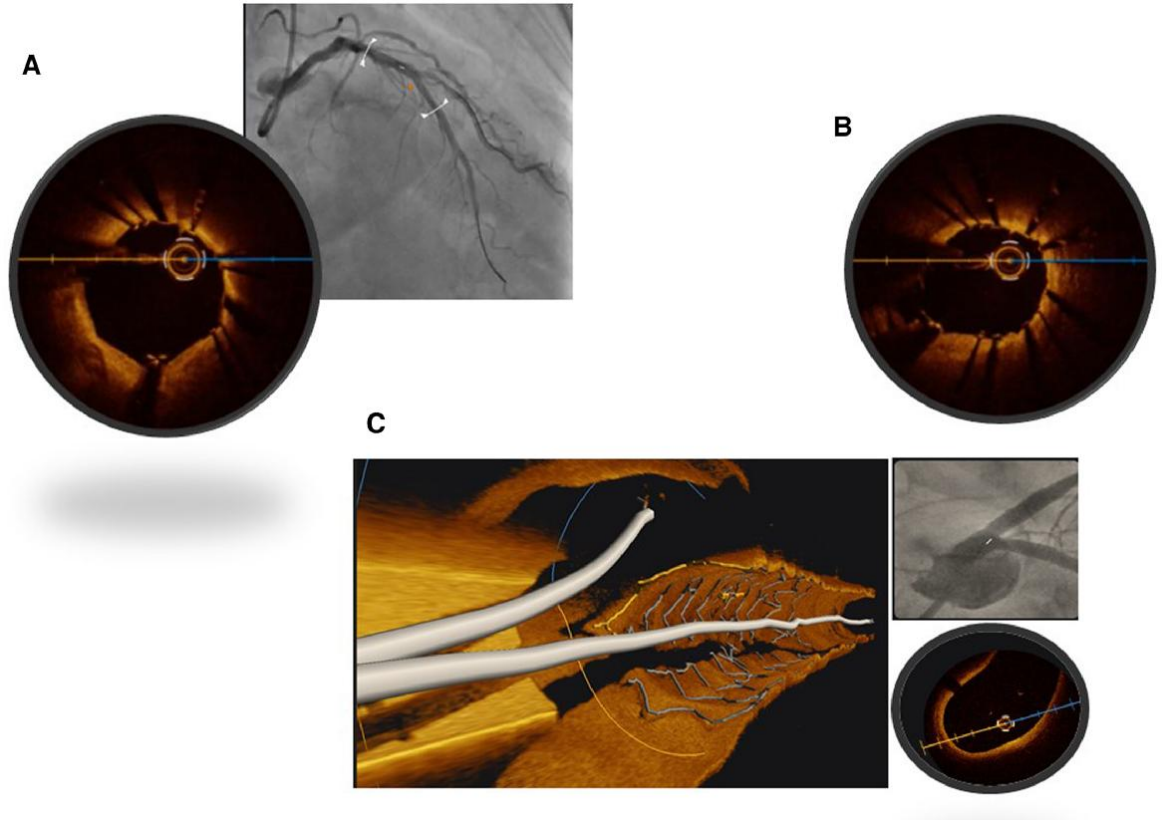
Jailed diagonal wire with provisional stenting strategy in LAD. D1 remains patent. (*A*) Proximal LAD stent before the bifurcation where the stent is well apposed and the D1 is jailed behind the LAD stent. (*B*) The bifurcation with diagonal wire comes from D1 ostium. Arrow indicates the jailed wire position. (*C*) 3D reconstruction of ostial LAD stent. Both lad and left circumflex wire are luminal.

Suboptimal stent expansion and malapposition have been associated with stent failure.^33^ Im and colleagues noted acute stent malapposition in 62% of lesions, with half of the acute stent malappositions located at stent edges. Severe diameter stenosis, calcified lesions, and long stents were independent predictors of acute stent malapposition. Late-acquired stent malapposition was identified in 15% of all lesions and was in the stent body in 61%. Late-acquired stent malapposition was more frequently associated with plaque/thrombus prolapse on post-stent OCT images (70% vs. 42%; *P* < 0.001). However, clinical events, including cardiovascular death, nonfatal MI, and ST, were not reported in patients with late stent malapposition.^33^ In order to avoid potentially devastating outcomes such as ST, intracoronary imaging during PCI is advocated to confirm landing zones and determine the success of pre-stent lesion preparation and optimize stent expansion post-stent (*Figure 3*). Several trials have required mid-procedure imaging in the trial design including the recently published OCTOBER Trial and the DK Crush VIII trial that is still recruiting.^10,34^ Furthermore, imaging can assist operators in discerning abluminal wiring and predicting side branch loss^11,35^ (*Figure 4*).

A comprehensive review of optimal best practices for use of IVI for pre-intervention lesion assessment, lesion preparation and stent deployment, and assessment of post-procedure endpoints and complications is beyond the scope of this manuscript. However, the recently published ILUMIEN IV trial analysed predictors of stent failure and identified an MSA < 4.2 mm^2^ as a major driver of TVF with a significantly elevated hazard. It also highlights the point that relative expansion becomes less relevant the larger the vessel treated.^8^

## Practice recommendations

Despite significant and accumulating data in favour of its use, IVI uptake remains low overall—with significant operator and centre variability.^28,36^ A growing evidence base of definitive clinical trials—which drove the recent updated 1A guideline recommendation for complex coronary lesion subsets—is expected to increase use. Additional barriers, to include knowledge gaps with image interpretation (and follow-on intervention to achieve optimal PCI endpoints) and lack of financial reimbursement, may also need to be better addressed before IVI can be fully incorporated into standard PCI workflow and cardiac catheterization laboratory culture.^37,38^ And although the evidence base for IVI has been primarily limited to trials in complex lesions subsets and it has been challenging to demonstrate significant benefits to IVI use in all comers (where the magnitude of benefit may be less due to low overall rates of adverse events for non-complex PCI in contemporary practice), universal use may help develop operator and centre expertise. An analysis at operator and institution level of the OCCUPI trial confirmed the expertise evident in East Asian operators.^17^

## Conclusion

Ample, and growing, evidence has demonstrated the benefit of intracoronary imaging in acute procedural and clinical outcomes. Recent guidelines have emphasized the critical role of intracoronary imaging—particularly in complex lesion subsets—and appropriately upgraded this to a class 1a recommendation in the most recent ESC guidelines. Professional societies, industry, and hospitals should together continue to enhance technologies and create user-friendly (and perhaps semi-automated) procedural schematics and algorithms and workflow enhancements to further promote uptake of IVI as part of a suite of best practices in the cardiac catheterization laboratory. Inclusion of IVI into reported performance metrics—now that there is significant evidence of mortality benefit—may also increase use and improve practice.

## Lead author biography

Dr Mirvat Alasnag is the Director of the Catheterization Laboratory at the King Fahd Armed Forces Hospital in Jeddah, Saudi Arabia. She was the first female interventional cardiologist in the Gulf region and has since represented women on multiple medical committees, including the Saudi Arabian Cardiac Interventional Society Board (current General Secretary) and the EAPCI Women's Steering Committee. She is the current ACCWIC International Working Group Chair and a SCAI WIN member.

Dr Alasnag trained in Clinical Cardiology and Interventional Cardiology at the Loma Linda University Medical Center (Loma Linda, CA) and Harper University Hospital, Detroit Medical Center (Detroit, MI), respectively. Since her graduation in 2009 to date, she has been a practicing interventional cardiologist at the King Fahd Armed Forces Hospital.

Dr Alasnag is currently serving as a member of the Board of Trustees for SCAI and a member of the ACC Interventional Leadership Council. Dr Alasnag is the Editor in Chief of *Current Cardiovascular Imaging Reports*. In addition, she is an Editor/Associate/Contributing Editor for *JSCAI*, *Circulation: Cardiovascular Interventions*, *PCROnline*, *Open Heart*, *Radcliffe Cardiology*, *ACCEL*, *Faculty Opinions* (ESC), and *Cardiovascular Revascularization Medicine*.

She is also a member of the ESC Congress Programme Committee, SCAI Scientific committee (Coronary Track), SCCT Annual Scientific Program committee, and EuroPCR Board. She is the Interventional Cardiology Section Chief of the Telemedicine Working Group and is an active on several other committees at ACC, SCAI, ESC, and SCCT including SCAI Board of Trustees and ACC Interventional Leadership Council. She is the nominal lead of the TCT Middle East Course Directors.

Dr Mirvat Alasnag is an editorial board member of *Interventional Cardiology: Reviews, Research, Resources (ICR3)* and *US Cardiology in Review, Radcliffe Cardiology*.
